# Draft Genome Sequence of a Taxonomically Unique *Neisseria* Strain Isolated from a Greater White-Fronted Goose (*Anser albifrons*) Egg on the North Slope of Alaska

**DOI:** 10.1128/genomeA.00772-15

**Published:** 2015-07-16

**Authors:** Cristina M. Hansen, Sang Chul Choi, Jayme Parker, Karsten Hueffer, Jack Chen

**Affiliations:** a Department of Biology and Wildlife, University of Alaska Fairbanks, Fairbanks, Alaska, USA; b Alaska State Public Health Virology Laboratory, Fairbanks, Alaska, USA; c Department of Veterinary Medicine, University of Alaska Fairbanks, Fairbanks, Alaska, USA

## Abstract

We report here the draft genome sequence of a unique *Neisseria* strain that was isolated from a greater white-fronted goose (*Anser albifrons*) egg. The sequencing was performed with an Illumina MiSeq system, and the sequence consists of 275 contigs. The total genome is 2,397,978 bp long and has a G+C content of 46.4%.

## GENOME ANNOUNCEMENT

The order *Neisseriales* consists of 32 genera, including the important pathogens *Neisseria gonorrhoeae* and *Neisseria meningitidis* (1). Other species of *Neisseriales* are sometimes isolated from human and animal specimens (2, 3). There are reports of *Neisseria* species being isolated from a sheldrake liver (4), the cloaca and phallus of domestic geese (5), and duck feces (6), and there is one report of *Neisseria animalis* being isolated from a mallard (*Anas platyrhynchos*) egg (7).

During the summer of 2013, we isolated a *Neisseria* species from the contents of 21 nonviable greater white-fronted goose (*Anser albifrons*) eggs on the North Slope of Alaska (8). We identified the DNA of this same species in an additional 2 nonviable eggs. Here, we report the draft genome sequence of a unique *Neisseria* strain (KH1503) isolated from the contents of an addled greater white-fronted goose egg. Based on our sequencing of the 16S rRNA gene and chaperonin 60 gene, this isolate seems to be most closely related to *Neisseria canis, Neisseria animaloris*, or *Neisseria shayeganii*. The identity scores for full-length (>1,400 bp) 16S rRNA gene sequences are ≤97%.

A pure culture was obtained by growing the isolate on blood agar plates at 37°. Bacteria were grown overnight in tryptic soy broth and pelleted by centrifugation at 5,000 × *g* for 10 min, and genomic DNA was extracted using a Wizard genomic DNA purification kit (Promega, Madison, WI). Genomic DNA was used to prepare a sequencing library using a Nextera DNA sample preparation kit (Illumina, San Diego, CA). Sequencing was performed on an Illumina MiSeq version 2 system and generated 24 to 30 million paired-end reads (2 × 250 bp), for a total of 7.4 Gbp.

We assembled the bacterial genome *de novo* using SPAdes version 2.5.1 (9). The assembled contigs totaled 2,397,978 bp. The assembly consisted of 275 contigs, ranging in size from 209 bp to 242,609 bp (median, 244 bp; mean, 8,720 bp) with a G+C content of 46.4%. We annotated the genome assembly using the NCBI Prokaryotic Genomes Annotation Pipeline (10). There are 2,334 putative genes, 2,159 coding sequences (CDSs), 122 pseudogenes, 3 rRNAs, 49 tRNAs, and 1 noncoding RNA. The genome sequence of this organism will allow for its further characterization and assessment of pathogen potential.

### Nucleotide sequence accession numbers. 

The draft genome sequence of *Neisseria* sp. KH1503 has been deposited at DDBJ/EMBL/GenBank under the accession no. JTDO00000000. The version described in this paper is version JTDO01000000.
