# Correction for Hansen et al., Draft Genome Sequence of a Taxonomically Unique *Neisseria* Strain Isolated from a Greater White-Fronted Goose (*Anser albifrons*) Egg on the North Slope of Alaska

**DOI:** 10.1128/genomeA.01775-15

**Published:** 2016-02-11

**Authors:** Cristina M. Hansen, Sang Chul Choi, Jayme Parker, Karsten Hueffer, Jack Chen

**Affiliations:** a Department of Biology and Wildlife, University of Alaska Fairbanks, Fairbanks, Alaska, USA; b Alaska State Public Health Virology Laboratory, Fairbanks, Alaska, USA; c Department of Veterinary Medicine, University of Alaska Fairbanks, Fairbanks, Alaska, USA

## AUTHOR CORRECTION

Volume 3, no. 4, e00772-15, 2015. Page 1: The byline should read as given above, and a present address footnote for Sang Chul Choi should be included.

